# Supplementary material for: Evaluation of the Virulence Potential of Listeria monocytogenes through the Characterization of the Truncated Forms of Internalin A
Source: Int J Mol Sci. 2023 Jun 14;24(12):10141. doi: 10.3390/ijms241210141 (PMC10299213; doi:10.3390/ijms241210141)
Supplement: Supplementary file 1 [file ijms-24-10141-s001.zip › Table S1.pdf]

Table S1. List of 849 *Listeria monocytogenes* strains with characterization (STs, lineages, and serotypes), sources, food origins and *inlA* type mutations.
nd: not detected. Serotypes still unknown because the STs are rarely and recently isolated.

STRAIN ID	ST	LINEAGE	SEROTYPE	SOURCE	FOOD ORIGIN	PMSC
1	**1**	I	4b	Food	Meat	Complete
2	**1**	I	4b	Food	Meat	Complete
3	**1**	I	4b	Food	Dairy	Complete
4	**1**	I	4b	Food	Dairy	Complete
5	**1**	I	4b	Food	Fish	Complete
6	**1**	I	4b	Food	Meat	Complete
7	**1**	I	4b	Food	Meat	Complete
8	**1**	I	4b	Clinical	/	Complete
9	**1**	I	4b	Clinical	/	Complete
10	**1**	I	4b	Clinical	/	Complete
11	**1**	I	4b	Clinical	/	Complete
12	**1**	I	4b	Clinical	/	Complete
13	**1**	I	4b	Clinical	/	Complete
14	**1**	I	4b	Clinical	/	Complete
15	**1**	I	4b	Clinical	/	Complete
16	**1**	I	4b	Clinical	/	Complete
17	**1**	I	4b	Clinical	/	Complete
18	**1**	I	4b	Food	Meat	Complete
19	**1**	I	4b	Food	Meat	Complete
20	**1**	I	4b	Food	Meat	Complete
21	**1**	I	4b	Food	Meat	Complete
22	**1**	I	4b	Food	Meat	Complete
23	**1**	I	4b	Food	Meat	Complete
24	**1**	I	4b	Food	Dairy	Complete
25	**1**	I	4b	Food	Meat	Complete
26	**1**	I	4b	Food	Meat	Complete
27	**1**	I	4b	Clinical	/	Complete
28	**1**	I	4b	Clinical	/	Complete
29	**1**	I	4b	Clinical	/	Complete
30	**1**	I	4b	Clinical	/	Complete
31	**1**	I	4b	Clinical	/	Complete
32	**1**	I	4b	Clinical	/	Complete
33	**1**	I	4b	Clinical	/	Complete
34	**1**	I	4b	Clinical	/	Complete
35	**1**	I	4b	Clinical	/	Complete
36	**1**	I	4b	Clinical	/	Complete
37	**1**	I	4b	Clinical	/	Complete
38	**1**	I	4b	Clinical	/	Complete
39	**1**	I	4b	Clinical	/	Complete
40	**1**	I	4b	Clinical	/	Complete
41	**1**	I	4b	Clinical	/	Complete
42	**1**	I	4b	Clinical	/	Complete
43	**1**	I	4b	Clinical	/	Complete
44	**1**	I	4b	Clinical	/	Complete
45	**1**	I	4b	Clinical	/	Complete
46	**1**	I	4b	Clinical	/	Complete

47	**1**	I	4b	Clinical	/	Complete
48	**1**	I	4b	Clinical	/	Complete
49	**1**	I	4b	Clinical	/	Complete
50	**1**	I	4b	Clinical	/	Complete
51	**1**	I	4b	Clinical	/	Complete
52	**1**	I	4b	Clinical	/	Complete
53	**1**	I	4b	Clinical	/	Complete
54	**1**	I	4b	Clinical	/	Complete
55	**1**	I	4b	Clinical	/	Complete
56	**1**	I	4b	Clinical	/	Complete
57	**1**	I	4b	Clinical	/	Complete
58	**1**	I	4b	Clinical	/	Complete
59	**1**	I	4b	Clinical	/	Complete
60	**1**	I	4b	Clinical	/	Complete
61	**1**	I	4b	Clinical	/	Complete
62	**1**	I	4b	Clinical	/	Complete
63	**1**	I	4b	Clinical	/	Complete
64	**1**	I	4b	Clinical	/	Complete
65	**1**	I	4b	Clinical	/	Complete
66	**1**	I	4b	Clinical	/	Complete
67	**1**	I	4b	Clinical	/	Complete
68	**1**	I	4b	Clinical	/	Complete
69	**1**	I	4b	Clinical	/	Complete
70	**1**	I	4b	Clinical	/	Complete
71	**1**	I	4b	Clinical	/	Complete
72	**1**	I	4b	Clinical	/	Complete
73	**1**	I	4b	Clinical	/	Complete
74	**1**	I	4b	Clinical	/	Complete
75	**1**	I	4b	Clinical	/	Complete
76	**2**	I	4b	Food	Other	Complete
77	**2**	I	4b	Food	Other	Complete
78	**2**	I	4b	Food	Dairy	Complete
79	**2**	I	4b	Food	Meat	Complete
80	**2**	I	4b	Clinical	/	Complete
81	**2**	I	4b	Clinical	/	Complete
82	**2**	I	4b	Clinical	/	Complete
83	**2**	I	4b	Food	Dairy	Complete
84	**2**	I	4b	Food	Dairy	Complete
85	**2**	I	4b	Food	Fish	Complete
86	**2**	I	4b	Food	Fish	Complete
87	**2**	I	4b	Food	Fish	Complete
88	**2**	I	4b	Food	Meat	Complete
89	**2**	I	4b	Food	Meat	Complete
90	**2**	I	4b	Food	Meat	Complete
91	**2**	I	4b	Environmental	/	Complete
92	**2**	I	4b	Environmental	/	Complete
93	**2**	I	4b	Environmental	/	Complete
94	**2**	I	4b	Environmental	/	Complete
95	**2**	I	4b	Environmental	/	Complete
96	**2**	I	4b	Environmental	/	Complete
97	**2**	I	4b	Environmental	/	Complete

98	**2**	I	4b	Environmental	/	Complete
99	**2**	I	4b	Environmental	/	Complete
100	**2**	I	4b	Clinical	/	Complete
101	**2**	I	4b	Clinical	/	Complete
102	**2**	I	4b	Clinical	/	Complete
103	**2**	I	4b	Clinical	/	Complete
104	**2**	I	4b	Clinical	/	Complete
105	**2**	I	4b	Clinical	/	Complete
106	**2**	I	4b	Clinical	/	Complete
107	**2**	I	4b	Clinical	/	PMSC 6
108	**2**	I	4b	Clinical	/	Complete
109	**2**	I	4b	Clinical	/	Complete
110	**2**	I	4b	Clinical	/	Complete
111	**2**	I	4b	Clinical	/	Complete
112	**2**	I	4b	Clinical	/	Complete
113	**2**	I	4b	Clinical	/	Complete
114	**2**	I	4b	Clinical	/	Complete
115	**2**	I	4b	Clinical	/	Complete
116	**2**	I	4b	Clinical	/	Complete
117	**2**	I	4b	Clinical	/	Complete
118	**2**	I	4b	Clinical	/	Complete
119	**2**	I	4b	Clinical	/	Complete
120	**3**	I	1/2b	Food	Other	Complete
121	**3**	I	1/2b	Food	Meat	Complete
122	**3**	I	1/2b	Environmental	/	Complete
123	**3**	I	1/2b	Environmental	/	Complete
124	**3**	I	1/2b	Clinical	/	Complete
125	**3**	I	1/2b	Food	Dairy	Complete
126	**3**	I	1/2b	Food	Dairy	Complete
127	**3**	I	1/2b	Food	Dairy	Complete
128	**3**	I	1/2b	Food	Meat	Complete
129	**3**	I	1/2b	Food	Meat	Complete
130	**3**	I	1/2b	Food	Dairy	Complete
131	**3**	I	1/2b	Food	Meat	Complete
132	**3**	I	1/2b	Food	Dairy	Complete
133	**3**	I	1/2b	Food	Meat	Complete
134	**3**	I	1/2b	Food	Meat	Complete
135	**3**	I	1/2b	Food	Meat	Complete
136	**3**	I	1/2b	Environmental	/	Complete
137	**3**	I	1/2b	Clinical	/	Complete
138	**3**	I	1/2b	Clinical	/	Complete
139	**3**	I	1/2b	Clinical	/	Complete
140	**4**	I	4b	Food	Dairy	Complete
141	**4**	I	4b	Clinical	/	Complete
142	**4**	I	4b	Clinical	/	Complete
143	**4**	I	4b	Clinical	/	Complete
144	**4**	I	4b	Clinical	/	Complete
145	**4**	I	4b	Clinical	/	Complete
146	**4**	I	4b	Clinical	/	Complete
147	**5**	I	1/2b	Food	Meat	Complete
148	**5**	I	1/2b	Food	Fish	Complete

149	5	I	1/2b	Food	Meat	Complete
150	5	I	1/2b	Food	Fish	Complete
151	5	I	1/2b	Food	Meat	Complete
152	5	I	1/2b	Food	Meat	Complete
153	5	I	1/2b	Food	Fish	Complete
154	5	I	1/2b	Food	Fish	Complete
155	5	I	1/2b	Food	Fish	Complete
156	5	I	1/2b	Food	Meat	Complete
157	5	I	1/2b	Food	Meat	Complete
158	5	I	1/2b	Food	Meat	Complete
159	5	I	1/2b	Food	Dairy	Complete
160	5	I	1/2b	Food	Meat	Complete
161	5	I	1/2b	Environmental	/	Complete
162	5	I	1/2b	Environmental	/	Complete
163	5	I	1/2b	Environmental	/	Complete
164	5	I	1/2b	Environmental	/	Complete
165	5	I	1/2b	Environmental	/	Complete
166	5	I	1/2b	Environmental	/	Complete
167	5	I	1/2b	Environmental	/	Complete
168	5	I	1/2b	Clinical	/	Complete
169	5	I	1/2b	Clinical	/	Complete
170	5	I	1/2b	Clinical	/	Complete
171	5	I	1/2b	Clinical	/	Complete
172	5	I	1/2b	Clinical	/	Complete
173	5	I	1/2b	Clinical	/	Complete
174	5	I	1/2b	Clinical	/	Complete
175	5	I	1/2b	Clinical	/	Complete
176	5	I	1/2b	Clinical	/	Complete
177	5	I	1/2b	Clinical	/	Complete
178	5	I	1/2b	Clinical	/	Complete
179	5	I	1/2b	Clinical	/	Complete
180	5	I	1/2b	Clinical	/	Complete
181	5	I	1/2b	Clinical	/	Complete
182	5	I	1/2b	Clinical	/	Complete
183	5	I	1/2b	Clinical	/	Complete
184	5	I	1/2b	Clinical	/	Complete
185	5	I	1/2b	Clinical	/	Complete
186	5	I	1/2b	Clinical	/	Complete
187	5	I	1/2b	Clinical	/	Complete
188	5	I	1/2b	Clinical	/	Complete
189	5	I	1/2b	Clinical	/	Complete
190	5	I	1/2b	Clinical	/	Complete
191	5	I	1/2b	Clinical	/	Complete
192	5	I	1/2b	Clinical	/	Complete
193	5	I	1/2b	Clinical	/	Complete
194	5	I	1/2b	Clinical	/	Complete
195	5	I	1/2b	Clinical	/	Complete
196	5	I	1/2b	Clinical	/	Complete
197	5	I	1/2b	Clinical	/	Complete
198	5	I	1/2b	Clinical	/	Complete
199	5	I	1/2b	Clinical	/	Complete

200	**5**	I	1/2b	Clinical	/	Complete
201	**5**	I	1/2b	Clinical	/	Complete
202	**5**	I	1/2b	Clinical	/	Complete
203	**5**	I	1/2b	Clinical	/	Complete
204	**5**	I	1/2b	Clinical	/	Complete
205	**5**	I	1/2b	Clinical	/	Complete
206	**5**	I	1/2b	Clinical	/	Complete
207	**5**	I	1/2b	Clinical	/	Complete
208	**5**	I	1/2b	Clinical	/	Complete
209	**5**	I	1/2b	Clinical	/	Complete
210	**5**	I	1/2b	Clinical	/	Complete
211	**5**	I	1/2b	Clinical	/	Complete
212	**5**	I	1/2b	Clinical	/	Complete
213	**5**	I	1/2b	Clinical	/	Complete
214	**5**	I	1/2b	Clinical	/	Complete
215	**5**	I	1/2b	Clinical	/	Complete
216	**5**	I	1/2b	Clinical	/	Complete
217	**5**	I	1/2b	Clinical	/	Complete
218	**5**	I	1/2b	Clinical	/	Complete
219	**5**	I	1/2b	Clinical	/	Complete
220	**5**	I	1/2b	Clinical	/	Complete
221	**5**	I	1/2b	Clinical	/	Complete
222	**5**	I	1/2b	Clinical	/	Complete
223	**5**	I	1/2b	Clinical	/	Complete
224	**5**	I	1/2b	Clinical	/	Complete
225	**5**	I	1/2b	Clinical	/	Complete
226	**5**	I	1/2b	Clinical	/	Complete
227	**5**	I	1/2b	Clinical	/	Complete
228	**5**	I	1/2b	Clinical	/	Complete
229	**5**	I	1/2b	Clinical	/	Complete
230	**5**	I	1/2b	Clinical	/	Complete
231	**5**	I	1/2b	Clinical	/	Complete
232	**6**	I	4b	Clinical	/	Complete
233	**6**	I	4b	Clinical	/	Complete
234	**6**	I	4b	Clinical	/	Complete
235	**6**	I	4b	Clinical	/	Complete
236	**6**	I	4b	Clinical	/	Complete
237	**6**	I	4b	Clinical	/	Complete
238	**6**	I	4b	Clinical	/	Complete
239	**6**	I	4b	Food	Meat	Complete
240	**6**	I	4b	Food	Fish	Complete
241	**6**	I	4b	Food	Dairy	Complete
242	**6**	I	4b	Food	Meat	Complete
243	**6**	I	4b	Food	Vegetable	Complete
244	**6**	I	4b	Food	Fish	Complete
245	**6**	I	4b	Clinical	/	Complete
246	**6**	I	4b	Clinical	/	Complete
247	**6**	I	4b	Clinical	/	Complete
248	**6**	I	4b	Clinical	/	Complete
249	**6**	I	4b	Clinical	/	Complete
250	**6**	I	4b	Clinical	/	Complete

251	**6**	I	4b	Clinical	/	Complete
252	**6**	I	4b	Clinical	/	Complete
253	**6**	I	4b	Clinical	/	Complete
254	**6**	I	4b	Clinical	/	Complete
255	**6**	I	4b	Clinical	/	Complete
256	**6**	I	4b	Clinical	/	Complete
257	**6**	I	4b	Clinical	/	Complete
258	**6**	I	4b	Clinical	/	Complete
259	**6**	I	4b	Clinical	/	Complete
260	**6**	I	4b	Clinical	/	Complete
261	**6**	I	4b	Clinical	/	Complete
262	**6**	I	4b	Clinical	/	Complete
263	**6**	I	4b	Clinical	/	Complete
264	**6**	I	4b	Clinical	/	Complete
265	**6**	I	4b	Clinical	/	Complete
266	**6**	I	4b	Clinical	/	Complete
267	**6**	I	4b	Clinical	/	Complete
268	**6**	I	4b	Clinical	/	Complete
269	**6**	I	4b	Clinical	/	Complete
270	**6**	I	4b	Clinical	/	Complete
271	**6**	I	4b	Clinical	/	Complete
272	**6**	I	4b	Clinical	/	Complete
273	**6**	I	4b	Clinical	/	Complete
274	**7**	II	1/2a	Food	Meat	Complete
275	**7**	II	1/2a	Food	Meat	Complete
276	**7**	II	1/2a	Environmental	/	Complete
277	**7**	II	1/2a	Environmental	/	Complete
278	**7**	II	1/2a	Environmental	/	Complete
279	**7**	II	1/2a	Environmental	/	Complete
280	**7**	II	1/2a	Clinical	/	Complete
281	**7**	II	1/2a	Food	Fish	Complete
282	**7**	II	1/2a	Food	Meat	Complete
283	**7**	II	1/2a	Clinical	/	Complete
284	**7**	II	1/2a	Clinical	/	Complete
285	**7**	II	1/2a	Clinical	/	Complete
286	**7**	II	1/2a	Clinical	/	Complete
287	**7**	II	1/2a	Clinical	/	Complete
288	**7**	II	1/2a	Clinical	/	Complete
289	**8**	II	1/2a	Food	Meat	Complete
290	**8**	II	1/2a	Food	Meat	Complete
291	**8**	II	1/2a	Food	Meat	Complete
292	**8**	II	1/2a	Environmental	/	Complete
293	**8**	II	1/2a	Environmental	/	Complete
294	**8**	II	1/2a	Clinical	/	Complete
295	**8**	II	1/2a	Clinical	/	Complete
296	**8**	II	1/2a	Clinical	/	Complete
297	**8**	II	1/2a	Clinical	/	Complete
298	**8**	II	1/2a	Clinical	/	Complete
299	**8**	II	1/2a	Clinical	/	Complete
300	**8**	II	1/2a	Food	Fish	Complete
301	**8**	II	1/2a	Food	Fish	Complete

302	8	II	1/2a	Food	Fish	Complete
303	8	II	1/2a	Food	Fish	Complete
304	8	II	1/2a	Food	Fish	Complete
305	8	II	1/2a	Food	Fish	Complete
306	8	II	1/2a	Food	Fish	Complete
307	8	II	1/2a	Food	Fish	Complete
308	8	II	1/2a	Food	Fish	Complete
309	8	II	1/2a	Food	Fish	Complete
310	8	II	1/2a	Food	Fish	Complete
311	8	II	1/2a	Food	Meat	Complete
312	8	II	1/2a	Food	Fish	Complete
313	8	II	1/2a	Food	Meat	Complete
314	8	II	1/2a	Food	Fish	Complete
315	8	II	1/2a	Food	Fish	Complete
316	8	II	1/2a	Food	Fish	Complete
317	8	II	1/2a	Food	Meat	Complete
318	8	II	1/2a	Food	Dairy	Complete
319	8	II	1/2a	Food	Meat	Complete
320	8	II	1/2a	Food	Fish	Complete
321	8	II	1/2a	Food	Meat	Complete
322	8	II	1/2a	Food	Meat	Complete
323	8	II	1/2a	Food	Fish	Complete
324	8	II	1/2a	Food	Meat	Complete
325	8	II	1/2a	Food	Meat	Complete
326	8	II	1/2a	Food	Fish	Complete
327	8	II	1/2a	Food	Meat	Complete
328	8	II	1/2a	Food	Meat	Complete
329	8	II	1/2a	Food	Fish	Complete
330	8	II	1/2a	Food	Fish	Complete
331	8	II	1/2a	Food	Fish	Complete
332	8	II	1/2a	Clinical	/	Complete
333	8	II	1/2a	Clinical	/	Complete
334	8	II	1/2a	Clinical	/	Complete
335	8	II	1/2a	Clinical	/	Complete
336	8	II	1/2a	Clinical	/	Complete
337	8	II	1/2a	Clinical	/	Complete
338	8	II	1/2a	Clinical	/	Complete
339	8	II	1/2a	Clinical	/	Complete
340	8	II	1/2a	Clinical	/	Complete
341	8	II	1/2a	Clinical	/	Complete
342	8	II	1/2a	Clinical	/	Complete
343	8	II	1/2a	Clinical	/	Complete
344	8	II	1/2a	Clinical	/	Complete
345	8	II	1/2a	Clinical	/	Complete
346	8	II	1/2a	Clinical	/	Complete
347	8	II	1/2a	Clinical	/	Complete
348	8	II	1/2a	Clinical	/	Complete
349	8	II	1/2a	Clinical	/	Complete
350	8	II	1/2a	Clinical	/	Complete
351	8	II	1/2a	Clinical	/	Complete
352	8	II	1/2a	Clinical	/	Complete

353	**8**	II	1/2a	Clinical	/	Complete
354	**8**	II	1/2a	Clinical	/	Complete
355	**8**	II	1/2a	Clinical	/	Complete
356	**8**	II	1/2a	Clinical	/	Complete
357	**9**	II	1/2c	Food	Meat	PMSC 19
358	**9**	II	1/2c	Food	Other	PMSC 11
359	**9**	II	1/2c	Food	Meat	Complete
360	**9**	II	1/2c	Food	Meat	PMSC 19
361	**9**	II	1/2c	Food	Meat	Complete
362	**9**	II	1/2c	Food	Meat	Complete
363	**9**	II	1/2c	Food	Meat	Complete
364	**9**	II	1/2c	Food	Fish	PMSC 19
365	**9**	II	1/2c	Food	Meat	PMSC 19
366	**9**	II	1/2c	Food	Other	PMSC 12
367	**9**	II	1/2c	Environmental	/	PMSC 13
368	**9**	II	1/2c	Environmental	/	PMSC 19
369	**9**	II	1/2c	Environmental	/	PMSC 11
370	**9**	II	1/2c	Food	Meat	PMSC 29
371	**9**	II	1/2c	Food	Meat	PMSC 29
372	**9**	II	1/2c	Food	Meat	PMSC 19
373	**9**	II	1/2c	Food	Meat	PMSC 29
374	**9**	II	1/2c	Food	Meat	PMSC 19
375	**9**	II	1/2c	Food	Fish	PMSC 29
376	**9**	II	1/2c	Food	Meat	PMSC 19
377	**9**	II	1/2c	Food	Meat	PMSC 11
378	**9**	II	1/2c	Food	Meat	PMSC 29
379	**9**	II	1/2c	Food	Meat	PMSC 19
380	**9**	II	1/2c	Food	Meat	PMSC 4
381	**9**	II	1/2c	Food	Meat	PMSC 4
382	**9**	II	1/2c	Food	Meat	PMSC 11
383	**9**	II	1/2c	Food	Meat	PMSC 26
384	**9**	II	1/2c	Food	Meat	PMSC 29
385	**9**	II	1/2c	Food	Meat	PMSC 19
386	**9**	II	1/2c	Food	Meat	PMSC 29
387	**9**	II	1/2c	Food	Meat	PMSC 29
388	**9**	II	1/2c	Food	Meat	PMSC 11
389	**9**	II	1/2c	Food	Meat	PMSC 29
390	**9**	II	1/2c	Food	Meat	PMSC 29
391	**9**	II	1/2c	Food	Meat	PMSC 29
392	**9**	II	1/2c	Food	Meat	PMSC 11
393	**9**	II	1/2c	Food	Meat	PMSC 29
394	**9**	II	1/2c	Food	Meat	PMSC 29
395	**9**	II	1/2c	Food	Meat	PMSC 29
396	**9**	II	1/2c	Food	Meat	PMSC 4
397	**9**	II	1/2c	Food	Meat	PMSC 4
398	**9**	II	1/2c	Food	Meat	PMSC 29
399	**9**	II	1/2c	Food	Meat	PMSC 29
400	**9**	II	1/2c	Food	Meat	PMSC 11
401	**9**	II	1/2c	Food	Meat	PMSC 29
402	**9**	II	1/2c	Food	Meat	PMSC 29
403	**9**	II	1/2c	Food	Fish	PMSC 4

404	**9**	II	1/2c	Food	Meat	PMSC 29
405	**9**	II	1/2c	Food	Meat	PMSC 29
406	**9**	II	1/2c	Food	Meat	PMSC 19
407	**9**	II	1/2c	Food	Meat	PMSC 29
408	**9**	II	1/2c	Food	Meat	PMSC 29
409	**9**	II	1/2c	Food	Meat	PMSC 29
410	**9**	II	1/2c	Food	Meat	PMSC 4
411	**9**	II	1/2c	Food	Meat	PMSC 19
412	**9**	II	1/2c	Food	Meat	PMSC 19
413	**9**	II	1/2c	Food	Meat	PMSC 29
414	**9**	II	1/2c	Food	Meat	PMSC 11
415	**9**	II	1/2c	Food	Meat	PMSC 8
416	**9**	II	1/2c	Food	Meat	PMSC 29
417	**9**	II	1/2c	Food	Meat	PMSC 29
418	**9**	II	1/2c	Food	Meat	PMSC 11
419	**9**	II	1/2c	Food	Meat	PMSC 29
420	**9**	II	1/2c	Food	Meat	PMSC 29
421	**9**	II	1/2c	Food	Meat	PMSC 29
422	**9**	II	1/2c	Food	Meat	PMSC 29
423	**9**	II	1/2c	Food	Meat	PMSC 29
424	**9**	II	1/2c	Food	Meat	PMSC 29
425	**9**	II	1/2c	Food	Meat	PMSC 4
426	**9**	II	1/2c	Food	Meat	PMSC 4
427	**9**	II	1/2c	Food	Meat	PMSC 4
428	**9**	II	1/2c	Food	Meat	PMSC 29
429	**9**	II	1/2c	Food	Meat	PMSC 29
430	**9**	II	1/2c	Food	Meat	PMSC 29
431	**9**	II	1/2c	Food	Meat	PMSC 29
432	**9**	II	1/2c	Food	Meat	PMSC 29
433	**9**	II	1/2c	Food	Meat	PMSC 11
434	**9**	II	1/2c	Food	Meat	PMSC 29
435	**9**	II	1/2c	Food	Meat	PMSC 29
436	**9**	II	1/2c	Food	Meat	PMSC 19
437	**9**	II	1/2c	Food	Meat	PMSC 29
438	**9**	II	1/2c	Food	Meat	PMSC 13
439	**9**	II	1/2c	Food	Meat	PMSC 11
440	**9**	II	1/2c	Food	Meat	PMSC 11
441	**9**	II	1/2c	Food	Meat	PMSC 29
442	**9**	II	1/2c	Food	Meat	PMSC 29
443	**9**	II	1/2c	Food	Meat	PMSC 29
444	**9**	II	1/2c	Food	Meat	PMSC 4
445	**9**	II	1/2c	Food	Meat	PMSC 29
446	**9**	II	1/2c	Food	Meat	PMSC 29
447	**9**	II	1/2c	Food	Vegetable	PMSC 11
448	**9**	II	1/2c	Food	Meat	PMSC 29
449	**9**	II	1/2c	Food	Meat	PMSC 11
450	**9**	II	1/2c	Food	Meat	PMSC 29
451	**9**	II	1/2c	Food	Meat	PMSC 11
452	**9**	II	1/2c	Environmental	/	PMSC 29
453	**9**	II	1/2c	Environmental	/	PMSC 19
454	**9**	II	1/2c	Environmental	/	PMSC 4

455	**9**	II	1/2c	Clinical	/	PMSC 19
456	**9**	II	1/2c	Clinical	/	PMSC 29
457	**9**	II	1/2c	Clinical	/	PMSC 29
458	**9**	II	1/2c	Clinical	/	PMSC 29
459	**9**	II	1/2c	Food	Meat	Complete
460	**9**	II	1/2c	Food	Fish	Complete
461	**9**	II	1/2c	Food	Fish	Complete
462	**9**	II	1/2c	Food	Fish	Complete
463	**9**	II	1/2c	Food	Fish	Complete
464	**9**	II	1/2c	Clinical	/	PMSC 29
465	**9**	II	1/2c	Clinical	/	PMSC 19
466	**9**	II	1/2c	Clinical	/	Complete
467	**14**	II	1/2a	Food	Dairy	Complete
468	**14**	II	1/2a	Food	Meat	Complete
469	**14**	II	1/2a	Clinical	/	Complete
470	**14**	II	1/2a	Food	Fish	Complete
471	**14**	II	1/2a	Food	Meat	Complete
472	**14**	II	1/2a	Food	Meat	Complete
473	**14**	II	1/2a	Food	Fish	Complete
474	**14**	II	1/2a	Clinical	/	Complete
475	**14**	II	1/2a	Clinical	/	Complete
476	**14**	II	1/2a	Clinical	/	Complete
477	**14**	II	1/2a	Clinical	/	Complete
478	**14**	II	1/2a	Clinical	/	Complete
479	**16**	II	1/2a	Food	Meat	Complete
480	**18**	II	1/2a	Food	Meat	Complete
481	**18**	II	1/2a	Food	Dairy	Complete
482	**18**	II	1/2a	Food	Meat	Complete
483	**18**	II	1/2a	Clinical	/	Complete
484	**18**	II	1/2a	Clinical	/	Complete
485	**18**	II	1/2a	Clinical	/	Complete
486	**18**	II	1/2a	Clinical	/	Complete
487	**18**	II	1/2a	Clinical	/	Complete
488	**18**	II	1/2a	Clinical	/	Complete
489	**19**	II	1/2a	Food	Fish	Complete
490	**20**	II	1/2a	Food	Meat	Complete
491	**20**	II	1/2a	Food	Vegetable	Complete
492	**20**	II	1/2a	Food	Meat	Complete
493	**20**	II	1/2a	Clinical	/	Complete
494	**21**	II	1/2a	Clinical	/	Complete
495	**21**	II	1/2a	Clinical	/	Complete
496	**26**	II	1/2a	Clinical	/	Complete
497	**26**	II	1/2a	Clinical	/	Complete
498	**26**	II	1/2a	Clinical	/	Complete
499	**26**	II	1/2a	Clinical	/	Complete
500	**29**	II	1/2a	Food	Meat	Complete
501	**29**	II	1/2a	Food	Dairy	Complete
502	**29**	II	1/2a	Clinical	/	Complete
503	**29**	II	1/2a	Clinical	/	Complete
504	**29**	II	1/2a	Clinical	/	Complete
505	**30**	II	1/2a	Food	Meat	Complete

506	**30**	II	1/2a	Food	Vegetable	Complete
507	**30**	II	1/2a	Clinical	/	Complete
508	**31**	II	1/2a	Food	Meat	PMSC 5
509	**31**	II	1/2a	Food	Meat	PMSC 5
510	**31**	II	1/2a	Food	Fish	PMSC 5
511	**31**	II	1/2a	Food	Meat	PMSC 5
512	**31**	II	1/2a	Food	Fish	PMSC 4
513	**32**	I	4b	Food	Meat	Complete
514	**32**	I	4b	Food	Other	Complete
515	**32**	I	4b	Clinical	/	Complete
516	**37**	II	1/2a	Food	Meat	Complete
517	**37**	II	1/2a	Food	Meat	Complete
518	**37**	II	1/2a	Food	Meat	Complete
519	**37**	II	1/2a	Food	Meat	Complete
520	**37**	II	1/2a	Environmental	/	Complete
521	**37**	II	1/2a	Environmental	/	Complete
522	**37**	II	1/2a	Clinical	/	Complete
523	**37**	II	1/2a	Clinical	/	Complete
524	**37**	II	1/2a	Food	Meat	Complete
525	**37**	II	1/2a	Food	Meat	Complete
526	**37**	II	1/2a	Food	Meat	Complete
527	**37**	II	1/2a	Food	Meat	Complete
528	**37**	II	1/2a	Environmental	/	Complete
529	**37**	II	1/2a	Clinical	/	Complete
530	**37**	II	1/2a	Clinical	/	Complete
531	**37**	II	1/2a	Clinical	/	Complete
532	**37**	II	1/2a	Clinical	/	Complete
533	**37**	II	1/2a	Clinical	/	Complete
534	**37**	II	1/2a	Clinical	/	Complete
535	**37**	II	1/2a	Clinical	/	Complete
536	**37**	II	1/2a	Clinical	/	Complete
537	**38**	II	1/2a	Clinical	/	Complete
538	**38**	II	1/2a	Food	Dairy	Complete
539	**38**	II	1/2a	Clinical	/	Complete
540	**38**	II	1/2a	Clinical	/	Complete
541	**38**	II	1/2a	Clinical	/	Complete
542	**38**	II	1/2a	Clinical	/	Complete
543	**38**	II	1/2a	Clinical	/	Complete
544	**38**	II	1/2a	Clinical	/	Complete
545	**38**	II	1/2a	Clinical	/	Complete
546	**54**	I	4b	Clinical	/	Complete
547	**59**	I	1/2b	Food	Meat	Complete
548	**59**	I	1/2b	Clinical	/	Complete
549	**59**	I	1/2b	Clinical	/	Complete
550	**59**	I	1/2b	Clinical	/	Complete
551	**59**	I	1/2b	Clinical	/	Complete
552	**87**	I	1/2b	Food	Vegetable	Complete
553	**87**	I	1/2b	Food	Fish	Complete
554	**87**	I	1/2b	Clinical	/	Complete
555	**87**	I	1/2b	Clinical	/	Complete
556	**87**	I	1/2b	Clinical	/	Complete

557	**101**	II	1/2a	Food	Fish	Complete
558	**101**	II	1/2a	Food	Dairy	Complete
559	**101**	II	1/2a	Clinical	/	Complete
560	**101**	II	1/2a	Clinical	/	Complete
561	**101**	II	1/2a	Clinical	/	Complete
562	**101**	II	1/2a	Clinical	/	Complete
563	**101**	II	1/2a	Clinical	/	Complete
564	**101**	II	1/2a	Clinical	/	Complete
565	**120**	II	1/2a	Clinical	/	Complete
566	**120**	II	1/2a	Clinical	/	Complete
567	**121**	II	1/2a	Food	Meat	PMSC 6
568	**121**	II	1/2a	Food	Meat	PMSC 6
569	**121**	II	1/2a	Food	Meat	PMSC 6
570	**121**	II	1/2a	Food	Meat	PMSC 6
571	**121**	II	1/2a	Food	Meat	PMSC 6
572	**121**	II	1/2a	Food	Fish	PMSC 6
573	**121**	II	1/2a	Food	Dairy	PMSC 6
574	**121**	II	1/2a	Environmental	/	PMSC 6
575	**121**	II	1/2a	Environmental	/	PMSC 6
576	**121**	II	1/2a	Environmental	/	PMSC 6
577	**121**	II	1/2a	Food	Fish	PMSC 6
578	**121**	II	1/2a	Food	Meat	PMSC 6
579	**121**	II	1/2a	Food	Fish	PMSC 6
580	**121**	II	1/2a	Food	Fish	PMSC 6
581	**121**	II	1/2a	Food	Fish	PMSC 6
582	**121**	II	1/2a	Food	Meat	PMSC 6
583	**121**	II	1/2a	Food	Fish	PMSC 6
584	**121**	II	1/2a	Food	Fish	PMSC 6
585	**121**	II	1/2a	Food	Fish	PMSC 6
586	**121**	II	1/2a	Food	Fish	PMSC 6
587	**121**	II	1/2a	Food	Meat	PMSC 6
588	**121**	II	1/2a	Food	Meat	PMSC 6
589	**121**	II	1/2a	Food	Meat	PMSC 6
590	**121**	II	1/2a	Food	Meat	PMSC 6
591	**121**	II	1/2a	Food	Fish	PMSC 6
592	**121**	II	1/2a	Food	Meat	PMSC 6
593	**121**	II	1/2a	Food	Fish	PMSC 6
594	**121**	II	1/2a	Food	Meat	PMSC 6
595	**121**	II	1/2a	Food	Fish	PMSC 6
596	**121**	II	1/2a	Food	Fish	PMSC 6
597	**121**	II	1/2a	Food	Meat	PMSC 6
598	**121**	II	1/2a	Food	Fish	PMSC 6
599	**121**	II	1/2a	Food	Meat	PMSC 6
600	**121**	II	1/2a	Food	Meat	PMSC 6
601	**121**	II	1/2a	Food	Fish	PMSC 6
602	**121**	II	1/2a	Food	Meat	PMSC 6
603	**121**	II	1/2a	Food	Meat	PMSC 6
604	**121**	II	1/2a	Food	Fish	PMSC 6
605	**121**	II	1/2a	Food	Fish	PMSC 6
606	**121**	II	1/2a	Food	Fish	PMSC 6
607	**121**	II	1/2a	Food	Meat	PMSC 6

608	**121**	II	1/2a	Food	Fish	PMSC 6
609	**121**	II	1/2a	Food	Fish	PMSC 6
610	**121**	II	1/2a	Food	Meat	PMSC 6
611	**121**	II	1/2a	Food	Meat	PMSC 6
612	**121**	II	1/2a	Food	Fish	PMSC 6
613	**121**	II	1/2a	Food	Fish	PMSC 6
614	**121**	II	1/2a	Food	Fish	PMSC 6
615	**121**	II	1/2a	Food	Dairy	PMSC 6
616	**121**	II	1/2a	Environmental	/	PMSC 6
617	**121**	II	1/2a	Environmental	/	PMSC 6
618	**121**	II	1/2a	Environmental	/	PMSC 6
619	**121**	II	1/2a	Environmental	/	PMSC 6
620	**121**	II	1/2a	Clinical	/	PMSC 6
621	**121**	II	1/2a	Food	Meat	Complete
622	**121**	II	1/2a	Food	Meat	Complete
623	**121**	II	1/2a	Clinical	/	PMSC 6
624	**121**	II	1/2a	Clinical	/	PMSC 6
625	**145**	I	4b	Environmental	/	Complete
626	**155**	II	1/2a	Food	Meat	Complete
627	**155**	II	1/2a	Environmental	/	Complete
628	**155**	II	1/2a	Clinical	/	Complete
629	**155**	II	1/2a	Clinical	/	Complete
630	**155**	II	1/2a	Clinical	/	Complete
631	**155**	II	1/2a	Clinical	/	Complete
632	**155**	II	1/2a	Food	Meat	Complete
633	**155**	II	1/2a	Food	Fish	Complete
634	**155**	II	1/2a	Food	Meat	Complete
635	**155**	II	1/2a	Food	Meat	Complete
636	**155**	II	1/2a	Food	Meat	Complete
637	**155**	II	1/2a	Food	Meat	Complete
638	**155**	II	1/2a	Food	Fish	Complete
639	**155**	II	1/2a	Food	Fish	Complete
640	**155**	II	1/2a	Food	Meat	Complete
641	**155**	II	1/2a	Food	Fish	Complete
642	**155**	II	1/2a	Food	Meat	Complete
643	**155**	II	1/2a	Food	Meat	Complete
644	**155**	II	1/2a	Food	Meat	Complete
645	**155**	II	1/2a	Clinical	/	Complete
646	**155**	II	1/2a	Clinical	/	Complete
647	**155**	II	1/2a	Clinical	/	Complete
648	**155**	II	1/2a	Clinical	/	Complete
649	**155**	II	1/2a	Clinical	/	Complete
650	**155**	II	1/2a	Clinical	/	Complete
651	**155**	II	1/2a	Clinical	/	Complete
652	**155**	II	1/2a	Clinical	/	Complete
653	**155**	II	1/2a	Clinical	/	Complete
654	**155**	II	1/2a	Clinical	/	Complete
655	**155**	II	1/2a	Clinical	/	Complete
656	**155**	II	1/2a	Clinical	/	Complete
657	**155**	II	1/2a	Clinical	/	Complete
658	**155**	II	1/2a	Clinical	/	Complete

659	**155**	II	1/2a	Clinical	/	Complete
660	**155**	II	1/2a	Clinical	/	Complete
661	**155**	II	1/2a	Clinical	/	Complete
662	**155**	II	1/2a	Clinical	/	Complete
663	**155**	II	1/2a	Clinical	/	Complete
664	**155**	II	1/2a	Clinical	/	Complete
665	**155**	II	1/2a	Clinical	/	Complete
666	**155**	II	1/2a	Clinical	/	Complete
667	**155**	II	1/2a	Clinical	/	Complete
668	**155**	II	1/2a	Clinical	/	Complete
669	**155**	II	1/2a	Clinical	/	Complete
670	**177**	II	1/2a	Clinical	/	Complete
671	**177**	II	1/2a	Clinical	/	Complete
672	**191**	I	1/2b	Clinical	/	Complete
673	**191**	I	1/2b	Clinical	/	Complete
674	**193**	II	1/2c	Food	Meat	PMSC 25
675	**199**	II	1/2a	Food	Dairy	PMSC 4
676	**199**	II	1/2a	Food	Dairy	PMSC 4
677	**200**	II	1/2a	Clinical	/	Complete
678	**204**	II	1/2a	Environmental	/	Complete
679	**204**	II	1/2a	Environmental	/	Complete
680	**204**	II	1/2a	Environmental	/	Complete
681	**204**	II	1/2a	Food	Fish	Complete
682	**206**	II	nd	Clinical	/	Complete
683	**206**	II	nd	Clinical	/	Complete
684	**206**	II	nd	Food	Meat	Complete
685	**206**	II	nd	Clinical	/	Complete
686	**207**	II	nd	Food	Meat	Complete
687	**213**	I	4b	Food	Meat	Complete
688	**217**	I	4b	Food	Dairy	Complete
689	**217**	I	4b	Environmental	/	Complete
690	**217**	I	4b	Environmental	/	Complete
691	**217**	I	4b	Clinical	/	Complete
692	**219**	I	4b	Clinical	/	Complete
693	**219**	I	4b	Food	Other	Complete
694	**219**	I	4b	Food	Meat	Complete
695	**219**	I	4b	Clinical	/	Complete
696	**219**	I	4b	Clinical	/	Complete
697	**219**	I	4b	Clinical	/	Complete
698	**219**	I	4b	Clinical	/	Complete
699	**224**	I	1/2b	Food	Meat	Complete
700	**224**	I	1/2b	Food	Meat	PMSC 29
701	**224**	I	1/2b	Food	Meat	Complete
702	**224**	I	1/2b	Food	Meat	Complete
703	**224**	I	1/2b	Food	Fish	Complete
704	**224**	I	1/2b	Food	Fish	Complete
705	**224**	I	1/2b	Food	Fish	Complete
706	**224**	I	1/2b	Food	Fish	Complete
707	**224**	I	1/2b	Food	Fish	Complete
708	**224**	I	1/2b	Food	Fish	Complete
709	**224**	I	1/2b	Food	Fish	Complete

710	**224**	I	1/2b	Food	Fish	Complete
711	**224**	I	1/2b	Food	Fish	Complete
712	**224**	I	1/2b	Food	Fish	Complete
713	**224**	I	1/2b	Food	Fish	Complete
714	**224**	I	1/2b	Food	Fish	Complete
715	**224**	I	1/2b	Food	Fish	Complete
716	**224**	I	1/2b	Food	Fish	Complete
717	**224**	I	1/2b	Food	Fish	Complete
718	**224**	I	1/2b	Food	Fish	Complete
719	**224**	I	1/2b	Food	Fish	Complete
720	**224**	I	1/2b	Food	Fish	Complete
721	**224**	I	1/2b	Food	Fish	Complete
722	**224**	I	1/2b	Food	Fish	Complete
723	**224**	I	1/2b	Food	Fish	Complete
724	**224**	I	1/2b	Food	Fish	Complete
725	**224**	I	1/2b	Food	Fish	Complete
726	**224**	I	1/2b	Food	Fish	Complete
727	**224**	I	1/2b	Food	Meat	Complete
728	**224**	I	1/2b	Clinical	/	Complete
729	**224**	I	1/2b	Clinical	/	Complete
730	**224**	I	1/2b	Clinical	/	Complete
731	**224**	I	1/2b	Clinical	/	Complete
732	**224**	I	1/2b	Clinical	/	Complete
733	**224**	I	1/2b	Clinical	/	Complete
734	**288**	I	1/2b	Food	Meat	Complete
735	**288**	I	1/2b	Environmental	/	Complete
736	**288**	I	1/2b	Food	Meat	Complete
737	**288**	I	1/2b	Clinical	/	Complete
738	**288**	I	1/2b	Clinical	/	Complete
739	**325**	II	1/2a	Environmental	/	PMSC 26
740	**325**	II	1/2a	Environmental	/	PMSC 26
741	**325**	II	1/2a	Food	Dairy	PMSC 26
742	**325**	II	1/2a	Environmental	/	PMSC 26
743	**325**	II	1/2a	Environmental	/	PMSC 26
744	**325**	II	1/2a	Environmental	/	PMSC 26
745	**325**	II	1/2a	Food	Dairy	PMSC 26
746	**325**	II	1/2a	Food	Dairy	PMSC 26
747	**325**	II	1/2a	Food	Dairy	PMSC 26
748	**325**	II	1/2a	Food	Dairy	PMSC 26
749	**325**	II	1/2a	Food	Meat	PMSC 26
750	**325**	II	1/2a	Environmental	/	PMSC 26
751	**325**	II	1/2a	Environmental	/	PMSC 26
752	**325**	II	1/2a	Food	Meat	PMSC 26
753	**325**	II	1/2a	Environmental	/	PMSC 26
754	**325**	II	1/2a	Environmental	/	PMSC 26
755	**325**	II	1/2a	Environmental	/	PMSC 26
756	**325**	II	1/2a	Food	Dairy	PMSC 26
757	**325**	II	1/2a	Environmental	/	PMSC 26
758	**325**	II	1/2a	Food	Dairy	PMSC 26
759	**325**	II	1/2a	Environmental	/	PMSC 26
760	**325**	II	1/2a	Food	Dairy	PMSC 26

761	**325**	II	1/2a	Food	Dairy	PMSC 26
762	**325**	II	1/2a	Environmental	/	PMSC 26
763	**325**	II	1/2a	Environmental	/	PMSC 26
764	**325**	II	1/2a	Environmental	/	PMSC 26
765	**325**	II	1/2a	Environmental	/	PMSC 26
766	**325**	II	1/2a	Environmental	/	PMSC 26
767	**325**	II	1/2a	Environmental	/	PMSC 26
768	**325**	II	1/2a	Environmental	/	PMSC 26
769	**325**	II	1/2a	Environmental	/	PMSC 26
770	**325**	II	1/2a	Environmental	/	PMSC 26
771	**325**	II	1/2a	Environmental	/	PMSC 26
772	**325**	II	1/2a	Environmental	/	PMSC 26
773	**325**	II	1/2a	Environmental	/	PMSC 26
774	**325**	II	1/2a	Environmental	/	PMSC 26
775	**325**	II	1/2a	Environmental	/	PMSC 26
776	**325**	II	1/2a	Environmental	/	PMSC 26
777	**325**	II	1/2a	Food	Dairy	PMSC 26
778	**325**	II	1/2a	Food	Dairy	PMSC 26
779	**325**	II	1/2a	Food	Dairy	PMSC 26
780	**325**	II	1/2a	Food	Dairy	PMSC 26
781	**325**	II	1/2a	Food	Dairy	PMSC 26
782	**325**	II	1/2a	Food	Dairy	PMSC 26
783	**325**	II	1/2a	Food	Dairy	PMSC 26
784	**325**	II	1/2a	Food	Dairy	PMSC 26
785	**325**	II	1/2a	Food	Dairy	PMSC 26
786	**325**	II	1/2a	Food	Dairy	PMSC 26
787	**325**	II	1/2a	Food	Dairy	PMSC 29
788	**325**	II	1/2a	Food	Dairy	PMSC 26
789	**325**	II	1/2a	Clinical	/	Complete
790	**325**	II	1/2a	Clinical	/	PMSC 26
791	**330**	I	1/2b	Food	Meat	Complete
792	**330**	I	1/2b	Food	Fish	Complete
793	**330**	I	1/2b	Environmental	/	Complete
794	**330**	I	1/2b	Food	Fish	Complete
795	**386**	II	nd	Food	Meat	Complete
796	**388**	I	4b	Food	Meat	Complete
797	**388**	I	4b	Clinical	/	Complete
798	**394**	II	1/2a	Food	Meat	Complete
799	**397**	I	nd	Food	Dairy	Complete
800	**397**	I	nd	Clinical	/	Complete
801	**398**	II	1/2a	Clinical	/	Complete
802	**398**	II	1/2a	Food	Meat	Complete
803	**398**	II	1/2a	Food	Dairy	Complete
804	**398**	II	1/2a	Clinical	/	Complete
805	**412**	II	nd	Food	Fish	Complete
806	**412**	II	nd	Food	Meat	Complete
807	**429**	I	1/2b	Food	Other	Complete
808	**429**	I	1/2b	Clinical	/	Complete
809	**431**	II	1/2a	Clinical	/	Complete
810	**451**	II	1/2a	Food	Dairy	Complete
811	**451**	II	1/2a	Environmental	/	Complete

812	**451**	II	1/2a	Clinical	/	Complete
813	**451**	II	1/2a	Clinical	/	Complete
814	**451**	II	1/2a	Clinical	/	Complete
815	**451**	II	1/2a	Clinical	/	Complete
816	**451**	II	1/2a	Clinical	/	Complete
817	**451**	II	1/2a	Clinical	/	Complete
818	**451**	II	1/2a	Clinical	/	Complete
819	**451**	II	1/2a	Clinical	/	Complete
820	**451**	II	1/2a	Clinical	/	Complete
821	**451**	II	1/2a	Clinical	/	Complete
822	**451**	II	1/2a	Clinical	/	Complete
823	**489**	I	1/2b	Environmental	/	Complete
824	**504**	II	1/2a	Food	Fish	Complete
825	**504**	II	1/2a	Clinical	/	Complete
826	**511**	II	nd	Clinical	/	Complete
827	**517**	I	4b	Clinical	/	Complete
828	**517**	I	4b	Food	Meat	Complete
829	**517**	I	4b	Clinical	/	Complete
830	**517**	I	4b	Clinical	/	Complete
831	**580**	II	1/2c	Food	Meat	PMSC 11
832	**580**	II	1/2c	Food	Meat	PMSC 11
833	**580**	II	1/2c	Food	Meat	PMSC 11
834	**580**	II	1/2c	Food	Meat	PMSC 11
835	**580**	II	1/2c	Food	Meat	PMSC 11
836	**580**	II	1/2c	Food	Meat	PMSC 11
837	**717**	II	1/2a	Environmental	/	PMSC 6
838	**717**	II	1/2a	Food	Fish	PMSC 6
839	**1247**	II	1/2a	Food	Fish	Complete
840	**1247**	II	1/2a	Clinical	/	Complete
841	**1247**	II	1/2a	Clinical	/	Complete
842	**1247**	II	1/2a	Clinical	/	Complete
843	**1247**	II	1/2a	Clinical	/	Complete
844	**1247**	II	1/2a	Clinical	/	Complete
845	**1584**	I	nd	Food	Fish	Complete
846	**2080**	I	4b	Clinical	/	Complete
847	**2687**	II	nd	Food	Meat	PMSC 11
848	**2687**	II	nd	Food	Meat	PMSC 11
849	**2687**	II	nd	Environmental	/	PMSC 11
